# In-vitro Antimicrobial Study of Non/irradiated Ylang-ylang Essential Oil Against Multi Drug Resistant Pathogens with Reference to Microscopic Morphological Alterations

**DOI:** 10.1007/s12088-023-01122-4

**Published:** 2023-11-10

**Authors:** Nora Mohamed Elkenawy, Mahmoud Abdel Wahab Soliman, Reham Rashad El-behery

**Affiliations:** 1 Drug Radiation Research Department, National Center for Radiation Research and Technology (NCRRT), Egyptian Atomic Energy Authority (EAEA), Cairo, Egypt; 2 Radiation Microbiology Department, National Center for Radiation Research and Technology (NCRRT), Egyptian Atomic Energy Authority (EAEA), Cairo, Egypt

**Keywords:** Ylang-ylang (YY), Essential oil (EO), GC–MS, Minimum inhibitory concentration (MIC), Scanning electron microscope (SEM), Gamma irradiation, Cytotoxicity

## Abstract

Essential oils have proven to possess great potential in the field of biomedicine due to their ability to effectively eradicate a diverse range of pathogenic microbes. In this study, the antimicrobial activity of ylang-ylang essential oil (YY-EO) was screened against twelve multidrug-resistant pathogens. The YY-EO was effective up to 536 μg/ml, with the highest inhibition zone in case of *S. aureus* MMCC21 and *Escherichia coli* MMCC24*.* The least effect on both *Bacillus cereus* MMCC11 *and Klebsiella pneumonia* MMCC16*.* The major components of the essential oil were identified using GC–MS analysis. Different gamma irradiation doses against the YY-EO were evaluated as a tool of natural decontamination. Moreover, the antimicrobial assay after irradiation proved no significant changes regarding the antimicrobial activity before and after irradiation of EO at the applied dose. The minimum inhibitory concentration (MIC) for the EO against the tested pathogens was detected. The possible morphological changes in some of the bacterial and yeast cells at the recognized MIC and 2MIC were detected using the scanning electron microscope (SEM). Results revealed a notable change in terms of both the microbial cell population and the morphology of the tested bacterial and yeast cells. The cytotoxicity of ylang-ylang EO was evaluated against normal skin tissue culture and showed a potential cytotoxic effect at concentrated doses. These results refer to the importance of YY-EO as a natural antimicrobial agent and the possible application of YY-EO as a surface decontaminant, but they also draw attention to the importance of the EO concentration used in different applications to avoid possible toxic effects.

**Supplementary Information:**

The online version contains supplementary material available at 10.1007/s12088-023-01122-4.

## Introduction

Researchers worldwide are in continuous frantic race to overcome or at least control infectious diseases. Antimicrobials are crucial in this race as a tool to eradicate different microbial infections. Nevertheless, continuous and prevalent emergence of multidrug resistant microbial strains continues to represent a huge health hazard facing researcher’s tremendous efforts in this field. Bacterial anti-microbial resistance (AMR) has emerged as one of the leading public health threats of the twenty-first century. The review on AMR, commissioned by the UK Government, argued that by 2050, AMR could be responsible for 10 million deaths per year [1]. The World Health Organization, WHO, and numerous other research groups agreed that the spread of AMR is an urgent issue requiring a global and coordinated action plan to address. The demand for novel therapies, preferably derived from natural sources, to avoid as much as possible the potential cytotoxic effects of traditional antibiotics has tremendously increased during the last decade.

Unfortunately, earlier record of the hasty, powerful resistance to newly applied antimicrobial agents suggests that those will also have a short lifespan [2]. Meanwhile, medicinal plants and the essential oil extracted from them, are of course among the best candidates for that job EOs, are of course among the best candidates for that job [3]. This could be due to their high concentration of bioactive compounds [4]

Essential oils are mainly volatile, liquid, lipid soluble, and organic solvents soluble. EOs may consist of 20–60 components with different concentrations. two or three major components exist at high concentrations (20–70%) in addition to other components present in trace amounts. The amount of the different components of essentials oils varies according to the different plant parts or plant species they are extracted from. They are chemically derived from terpenes and their oxygenated derivatives i.e. terpenoids [5]

One main characteristic of essential oils and their components is hydrophobicity, which allows them to partition with the lipids present in the bacterial cell membrane and mitochondria, hence, rendering them more permeable through disturbing the cell structures. Eventually, that results in the death of bacterial cell due to leakage of vital molecules and ions from the bacterial cell. Some compounds even modulate drug resistance by targeting efflux mechanisms of some Gram-negative bacteria [6]

EOs have been effectively employed for the treatment of microbial infections and related disorders for centuries due to their potent antibacterial, antifungal and antiviral properties [7]. Including urinary tract infections [8] in addition to intestinal and respiratory disorders [9]. In addition, some EOs are employed as a main and active ingredient in cosmetics, disinfectants as well as preserving food [10, 11].

Ylang-ylang, or *C. odorata,* is a tree known for its fragrant flower, natively present in Asian nations, with a pleasant floral aroma similar to jasmine. EO, extracted from the YY petals using steam distillation, represents an essential raw material for a considerable number of industries, including but not limited to the fragrance, cosmetics, and food industries [12].

A blended essential oil preparation prepared by [13] encountering a variety of essential oils (lavender, clary sage, ylang ylang, and jasmine) at different ratios exhibited antibacterial activity against some isolated strains of Gram-positive bacteria as *Staphylococcus aureus* ATCC6538 and *Staphylococcus epidermidis* and Gram-negative bacterium *Escherichia coli* ATCC25922, in addition to antifungal activity against *Candida albicans* ATCC10231.

EOs extraction from plants usually confers microbial contamination that occurs due to processing, handling and packaging which affects the integrity of the final product [14].

Commonly used thermal methods for the microbial decontamination of EOs usually affect the oil, which is reflected in the loss of aroma or chemical modification in the oil’s profile [15].

On the other hand, gamma radiation provides an alternative effective method for reducing or eliminating microbial contamination of EOs in a heat free manner and without altering their chemical composition if used at the right dose. Gamma radiation can also increase the total phenols, total flavonoids and total antioxidants of some herbs at doses below 5 kGy [16].

Most EOs are safe, with no considerable side effects when properly used [17], 18. However, since they are introduced on or into our bodies, they have to be used wisely. Accordingly, one must pay attention to some factors like dosage, concentration, purity, application method, or even possible drug interactions while applying essential oils.

Recently, studies of eukaryotic cell lines have indicated that EOs can possess a pro-oxidant and cytotoxic action. Therefore, an evaluation of their cytotoxicity is always required for the effective clinical use [19]. Accordingly, some EOs have been found to be harmful to the skin, liver, and other organs when used incorrectly or in high concentrations. However, other oils are considered safe for inhalation but may be irritating if applied to the skin, like: thyme, oregano, clove, and cinnamon. Several citrus oils, such as bergamot, lemon, lime, orange, and angelica, can be safely inhaled but can cause phototoxicity (severe burns or skin cancer) if exposed to natural sunlight or sunbed radiation following skin applications.

This study was conducted to examine a commercially available YY-EO in terms of its aromatic profile, antimicrobial activity against various multi drug resistant strains, cytotoxicity, and the role of gamma radiation as a possible tool for decontaminating EOs.

## Materials and Methods

### Materials

Ylang-ylang (*C. odorata*) EO of analytical grade was purchased from Oil House Egyptian company https://www.oilhouse-eg.com/. Nutrient agar (NA), nutrient broth (NB), Sabaroud dextrose broth(SDB), Sabaroud dextrose agar (SDA) (Oxoid, UK), Agar (CAS 9002-18-0) and DMSO CAS 67-68-5 (Sigma Aldrich, UK).

### Volatiles’ Profile of YY-EO

Volatiles’ profile of YY-EO was performed using GC–MS using (Agilent Technologies 7890B GC Systems combined with 5977A Mass Selective Detector). Capillary column (HP-5MS Capillary; 30.0 m × 0.25 mm ID × 0.25 μm film) was used and the carrier gas was helium at a pressure of 8.2 psi with 1 μl injection. The sample was analyzed with the column held initially for 3 min at 60 °C after injection, then the temperature was increased to 300 °C with a 15 °C/min heating ramp, with a 3.0 min hold. Injection was carried out in split mode with a ratio (1:1) at 300 °C. MS scan range was (*m*/*z*): 50–550 atomic mass units (AMU) under electron impact (EI) ionization (70 eV) and solvent delay 3.0 min. The constituents were determined by mass fragmentations with The NIST mass spectral search program for the NIST/EPA/NIH mass spectral library Version 2.2 [20].

### Microorganisms

Twelve isolates from environmental and clinical sources were supplied by the medical microbiology lab culture collection (MMCC) of the Drug Radiation Research Department in the (NCRRT). Four Gram Positive *Bacillus cereus* MMCC11*, Bacillus cereus* MMCC12, *Staphylococcus aureus* MMCC21 and *Staphylococcus aureus* MMCC22*,* six Gram Negative *Pseudomonas aeruoginosa* MMCC13*, Pseudomonas aeruoginosa* MMCC14*, Echerichia coli* MMCC24*, Echerichia coli* MMCC25*, Klebsiella pneumonia* MMCC16 and *Klebsiella pneumonia* MMCC17 and two yeasts *Candida tropicalis* MMCC12 and *Candida parapsilosis* MMCC24*.* All isolates were previously identified following standard biochemical tests and confirmed using VITEK 2 Systems Version: 08.01 at the Chemical Warfare Laboratories and MALDI-TOF (SAI) in the National center for Radiation Research Technology(NCRRT).

### Antimicrobial Evaluation of YY-EO

The antimicrobial activity of YY-EO was determined using the agar-well diffusion method as described by [21] with slight modifications. Prior to the experiment, a single colony was inoculated in NB for bacterial isolates and incubated at 37 °C for 24 h, and in SDB for yeast isolates and incubated at 28 °C for 48 h. Overnight microbial culture was spectrophotometrically at wavelength 600 nm adjusted to 10^8^ cfu/ml to be used in experimental procedures.

Nutrient agar plates for bacteria and SDA for yeasts were inoculated with 100 μl of overnight microbial suspension was swabbed using a sterile swab and allowed to dry on the agar surface. Subsequently, wells of 6 mm diameter were punched into the agar medium using a cork borer and filled with 100 μl of YY-EO which was allowed to diffuse at room temperature for 20 min. At the same time some commonly prescribed antimicrobials discs were used as positive control (Ciprofloxacin (5 μg), Amikacin (30 μg)*,* Amoxicillin/ Clavulanic acid (30 μg) and Trimethoprim/Sulfamethoxazole (25 μg) for bacteria and Nystatin (100 IU) for yeast.

The plates were then incubated at 37 °C for 24 h for bacteria and incubated at 28 °C for 48 h for yeast. After incubation, the diameters of the growth inhibition zones were measured in mm. Data points were calculated from the mean of duplicates and expressed as mean ± standard deviation.

### Minimum Inhibitory Concentration of YY-EO

The antimicrobial potency of YY-EO was evaluated by means of the minimum inhibitory concentrations (MICs) using the broth microdilution modification method published by the Clinical and Laboratory Standards Institute CLSI 2012 [22]for bacteria and Clinical and Laboratory Standards Institute CLSI M 27-A2 for yeasts [23].The MIC was identified as the minimum concentration of drug for the first well with no visible growth compared to the oil-free well. For assays, the YY-EO was dissolved in DMSO and serial dilutions were prepared (starting from 7.8125 to 4000 μg/ml), In a 96 well plate, each EO concentration was inoculated into a column of wells filled with the bacterial or yeast adjusted suspension, then incubated at 37 °C for 24 h for bacteria and 28 °C for 48 h for yeast. Data points were calculated from the mean of duplicates and expressed as mean ± standard deviation.

### Effect of Gamma Irradiation on YY-Eo

YY-EO was irradiated using different doses of gamma irradiation (0.5, 1, 1.5 and 6 kGy) using a ^60^Co-source Indian gamma cell (GE 4000A) located at the National Center of Radiation and Research Technology (NCRRT), Egyptian Atomic Energy Authority (EAEA), Egypt. The dose rate was 1 kGy/h at the time of experiment.

#### Attenuated Total Reflectance-Fourier Transform Infrared Spectroscopy (FTIR)

Attenuated Total Reflectance-Fourier Transform Infrared spectroscopy (ATR-FTIR) was employed to detect any alteration that might have taken place at the YY-EO s functional groups or chemical composition due to exposure to gamma irradiation decontamination doses. Samples were scanned within the range of 4000–400 cm^−1^ using BRUKER VERTEX 70 device at NCRRT. The spectrum obtained was compared to literature.

#### Reassessment of Antimicrobial Activity After Gamma Irradiation of YY-EO

The antimicrobial activity of gamma-irradiated YY-EO was reassessed against the susceptible microorganisms to detect any alterations in the antimicrobial activity of the YY-EO as a result of exposure to gamma irradiation.

### Scanning Electron Microscopy

The effect of YY-EO on the morphology of the most susceptible tested strains was studied at concentrations of MIC and 2MIC, respectively; untreated strains were set as control using SEM. Microbial samples were prepared for electron microscopy according to the recommended method [24] and imaged using Scanning Electron Microscope Zeiss, EVO 15 (Germany) at the National Center for Radiation Research and Technology (NCRRT), Cairo, Egypt. SEM images were recorded at 25 kV magnifications.

### Cytoxicity assay of YY-EO

Cytotoxic effect of YY-EO against normal skin cells HFB4 cells purchased from the Holding Company for Biological Products & Vaccines (VACSERA) Giza, Egypt was done using method of functional assay of viability and cytotoxicity) (MTT) [25–27] We worked according to the Guidelines for the use of cell lines in biomedical research https://www.ncbi.nlm.nih.gov/pmc/articles/PMC4453835/#:~:text=Patient%20consent%20is%20usually%20required,%2Dtransfer%20agreement%20(MTA).

### Statistical Analysis

Data were expressed as mean ± standard error (SE). GraphPad Prism® software program was used for analysis, one-way analysis of variance (ANOVA) test followed by Tukey- Kramer multiple comparison’s test was selected to carry out all statistical tests.

## Results

We employed GC/MS analysis to account for volatiles’ profile composition in tested YY. Results are depicted in Table 1S, and a chromatogram showing compound peaks was provided as supplementary Fig. 1S, revealing 31 compounds, with monoterpene linalool (27.73%), phenolylpropanoids benzyl acetate (22.76%), tricyclic sesquiterpene α-Gurjunene (17.69%), and monoterpene linalyl acetate (7%) dominating the highest contribution percentage.

**Fig. 1:**
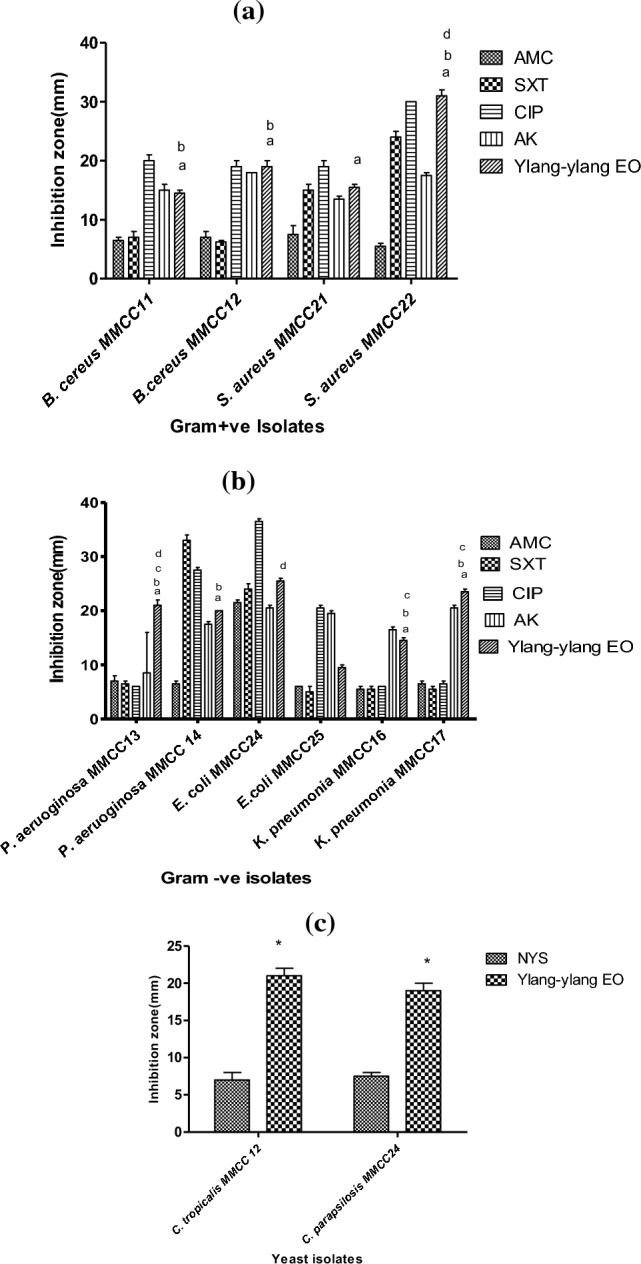
Antimicrobial activity of YY-EO against twelve strains (**A**) Gram + ve (**B**) Gram  − ve (**C**) yeast in comparison to commonly used antimicrobials. (a) indicates significance against AMC, (b) indicates significance against SXT, (c) indicates significance against CIP, (d) indicates significance against AK and (*) indicates significance against NYS

YY-EO antimicrobial potential was challenged against twelve Gram positive and Gram negative environmental and clinical isolates as well as yeasts using the agar well diffusion method. Results revealed its potent variable antimicrobial effect towards all tested strains, with the highest inhibition in the case of S. aureus MMCC21 (31 mm) and the least effect towards B. cereus MMCC11 (14.5 mm). Figure 1A shows that E. coli MMCC24 (25.5 mm) was the most susceptible Gram –ve isolate and K. pneumonia MMCC16 was the least susceptible (Fig. 1B). YY-EO zones of inhibition obtained from the antimicrobial susceptibility testing were statistically compared to those of commonly prescribed antimicrobials. YY-EO also expressed an even better antimicrobial effect than the most potent antibiotic, ciprofloxacin, in the cases of S. aureus MMCC22 and K. pneumonia MMCC17.In case of yeasts, YY-EO was much superior to the most commonly used antifungal nystatin Fig. 1C

We selected the most susceptible strains: Gram positive (*S. aureus* MMCC22), Gram negative (*E. coli* MMCC24), and yeast *(C. tropicalis* MMCC12) to pursue the study. Minimum inhibitory concentration results uncovered that YY-EO was capable of inhibiting the growth of *S. aureus* MMCC22*, E. coli* MMCC24, and *C. tropicalis* MMCC12 up to 500 μg/ml.

**Fig. 2:**
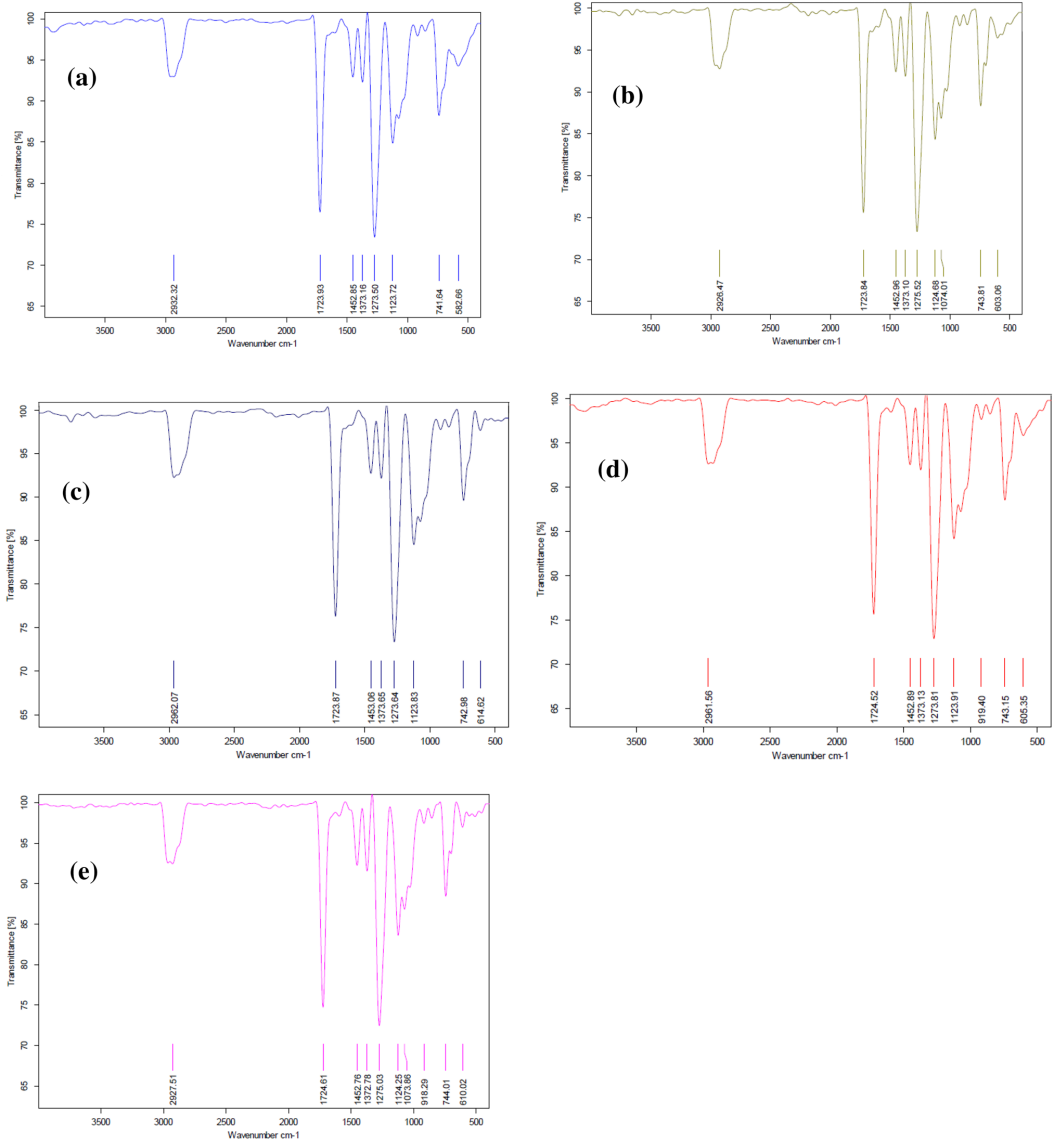
FTIR of YY-EO **a** un-irradiated, **b** 0.5 kGy, **c** 1 kGy, **d** 1.5 kGy and **e** 6 kGy

Variable doses of gamma radiation were employed to assess the effect on constituents present in the oil. Gamma radiation's effect was studied using low doses (0.5, 1, and 1.5 kGy) and a decontamination dose of 6 kGy. The effect of gamma radiation was reassessed using FTIR to look for chemical alterations represented by peak changes when compared to the control (un-irradiated) Fig. 2.

Peaks appearing in area of 400—1724 cm^−1^ known as “finger print region” exhibited no change in all tested doses. However, results of FTIR spectrums of both non-irradiated and irradiated EO at different doses (0.5, 1, 1.5 and 6 kGy) illustrated in Fig. 2.exhibited one broad peak at wave number 2932, 2926, 2962, 2961 and 2927 cm^−1^ respectively which assigned functional group C-H stretching- Alkaline.

**Fig. 3:**
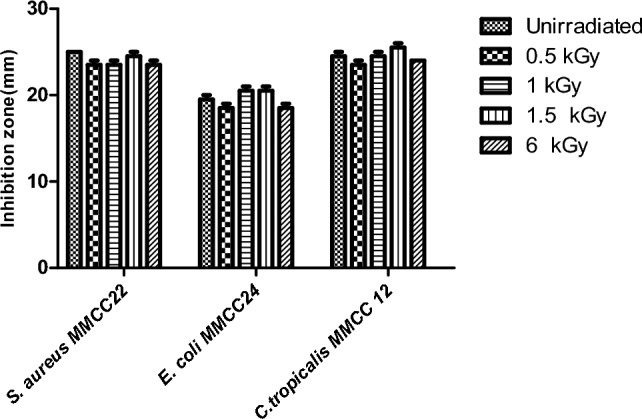
Antimicrobial evaluation unirradiated and irradiated YY-EO at doses (0.5, 1, 1.5 and 6 kGy) against *S. aureus* MMCC2 2*, E coli* MMCC 24 and *C tropicalis* MMCC12

**Fig. 4:**
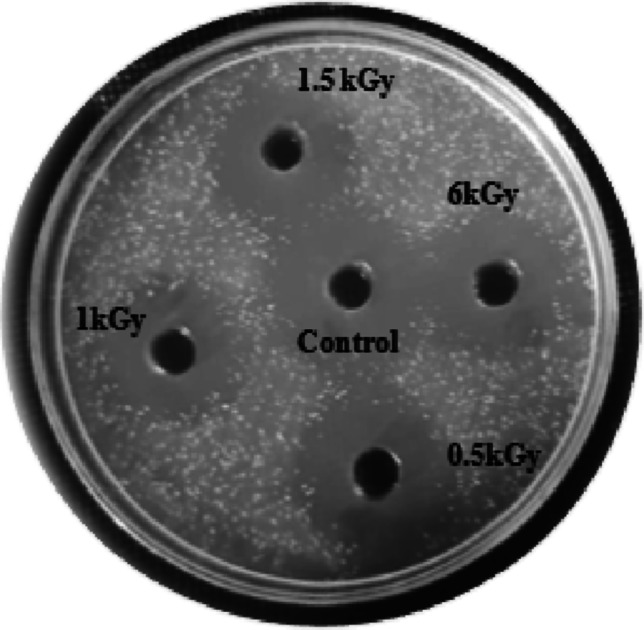
Antimicrobial evaluation of irradiated YY-EO at doses (0.5, 1, 1.5 and 6 kGy) vs control (un irradiated) against *S. aureus* MMCC 22

Antimicrobial potential of YY-EO was re-evaluated after varying levels of radiation. The results in Fig. 3 reveal that there were no substantial changes in the activity of the EO as the oil remained powerful against the tested strains, as seen in Fig. 4 against *S. aureus* MMCC 22.

In this study the direct antimicrobial effect of YY-EO on the cellular morphology was evaluated at MIC and 2MIC on the most susceptible Gram positive, Gram negative and *Candida* strains. The results from SEM imaging generally exhibited variations in cell morphology and decline in cells population.

**Fig. 5:**
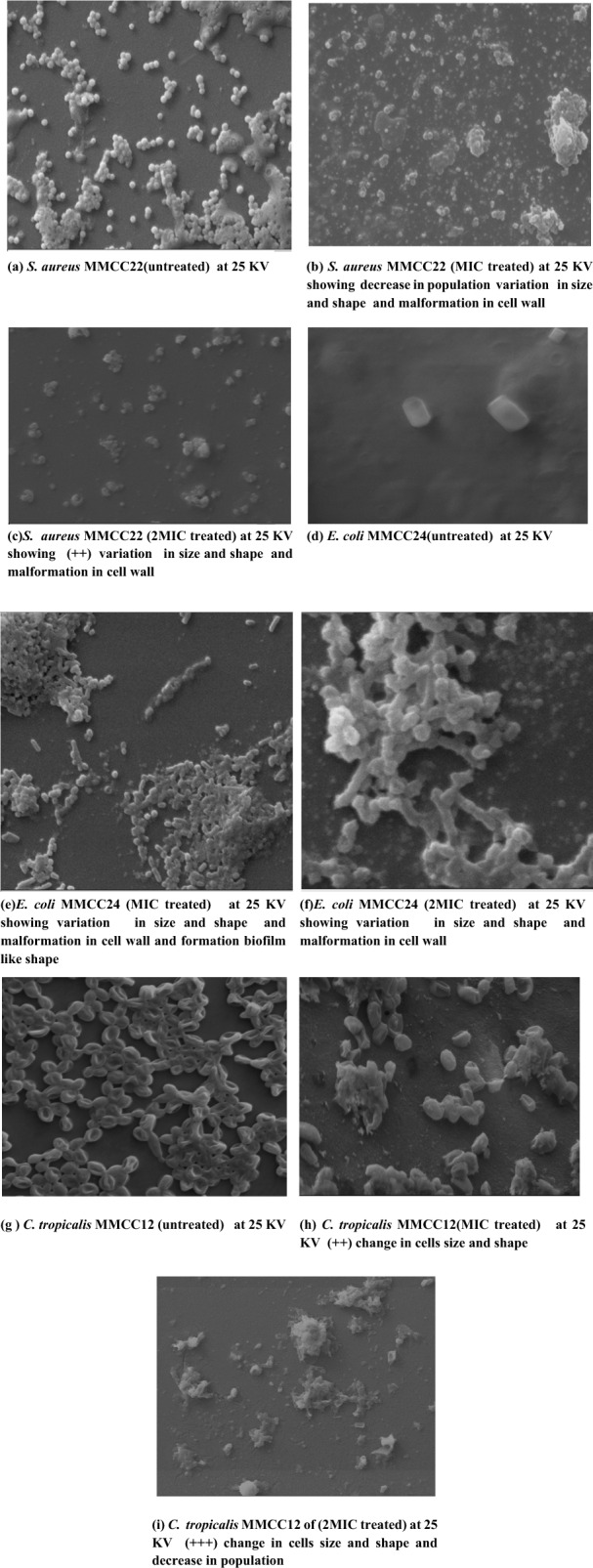
Morphological assessment of Ylang–ylang EO effect on treated at MIC and 2 MIC) on *S. aureus* MMCC22 (**a**, **b** and **c**), *E.coli* MMCC24 (**d**, **e** and **f**) and *C.tropicalis* MMCC12 (**g**, **h** and **i**) (untreated control)

Scanning electron microscope of *S. aureus* MMCC22 (untreated) at 25 kV shown in Fig. 5a revealed spherical, regular and intact cells and with smooth surface, whilst Fig. 5b and c at MIC and 2MIC respectively, exhibited marked deformation in cell morphology and decline in cells population.

In addition, Fig. 5d depicted a normal cell of *E. coli* MMCC 24 (untreated) with distinguishing traits such as a regular rod-shaped, undamaged surface, and striated cell walls. In contrast, most of the treated bacteria became irregular and shrivelled at MIC and 2MIC, suggesting the creation of cell aggregation (Biofilm-like shape) and modification of cell shape and size. Furthermore, cell walls of bacteria treated at 2MIC revealed more severe morphological damage than those treated at MIC.

Figure 5g, on the other hand, showed typical cell morphology displaying budding of *C. tropicalis* MMCC12, whereas Fig. 5h with MIC exhibited modest deformation in cell shape, and Fig. 5i transmitting 2MIC revealed a significant alteration in *C. tropicalis* cell size and count.

**Fig. 6:**
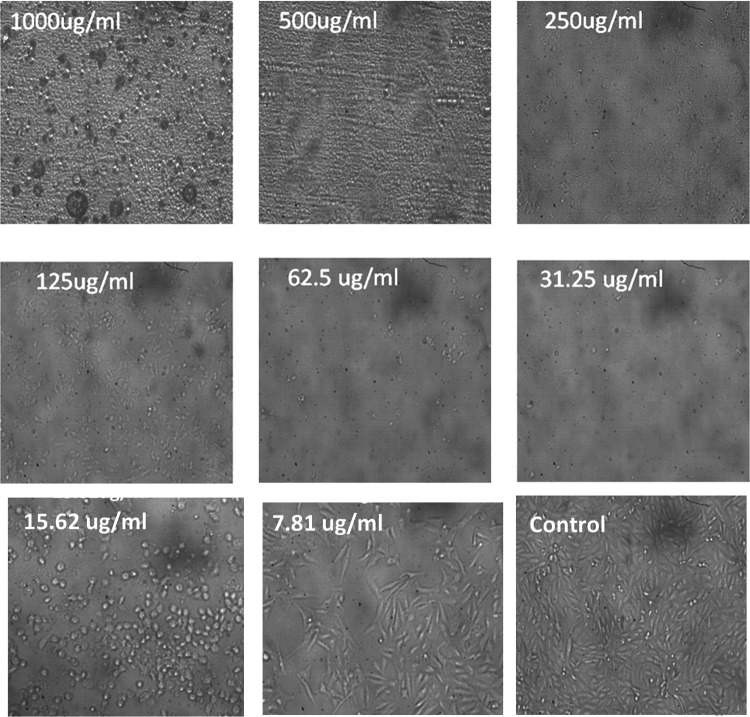
Effect of YY-EO at different concentrations on HBF4 normal skin cells

Cytoxicity assay of YY-EO on HFB4 normal skin cells at concentration 1000 μg/ml exhibited highly toxic effect on cells with only 6.06% viability (Fig. 6).

Increase in viability started to reappear at 15.62 μg/ml Table 1.

**Table1:** Effect of YY-EO on viability of HFB4 skin cells

ID	ug/ml	Mean O.D	± SE	Viability %	Toxicity %	IC50 ± SD
HFB4	–	0.758	0.004726	100	0	μg
1	1000	0.046	0.005196	6.068601583	93.93139842	9.7 ± 0.56
	500	0.039	0.001528	5.145118734	94.85488127	
	250	0.019	0.000577	2.506596306	97.49340369	
	125	0.018333	0.000667	2.418645558	97.58135444	
	62.5	0.019667	0.000333	2.594547054	97.40545295	
	31.25	0.020333	0.001202	2.682497801	97.3175022	
	15.62	0.147333	0.009939	19.43711522	80.56288478	
	7.81	0.450333	0.009735	59.41072999	40.58927001	

## Discussion

The use of EOs known for possessing antimicrobial phytochemicals, is usually perceived as a novel antimicrobial source. Although many essential oils from different aromatic plants have been investigated, the YY-EO potentials is still under research.

The medicinal properties exhibited by YY-EO are one of the main factors contributing to its increasing popularity. YY-EO has been previously approved for use in food by the FDA; the Flavour and Extract Manufacturers Association (FEMA) and the International Organization of Flavour Industries (IOFI) judged it to be safe for ingestion [28].

Y-EO was tested against twelve different pathogenic strains to evaluate its antimicrobial potential against various Gram-positive and Gram-negative pathogens as well as yeasts using the agar-well diffusion method. Results indicated considerable antimicrobial activity against most of the tested pathogenic strains.

Many phytochemical studies have identified the constituents present in the essential oils of *C. odorata*. A wide range of chemical compounds including monoterpene, sesquiterpenes, and phenylpropanoids have been isolated from this plant [29].However, various factors can affect the essential oil volatile profile, including geographical origin, climate, and seasonal variations [30].

Aromatic profile of YY tested oil conducted at this study detected thirty one volatile compounds in the tested oil present at variable percentages, with linalool as the chief component comes in agreement with [29]. Linalool is a well-known volatile monoterpene constituent present in many flowers and spices, contributing in the antimicrobial characteristics of EOs as seen with lavender oil [31]. Linalool (3,7-dimethyl-octa-1,6-diene-3-ol), a terpenic alcohol belonging to monoterpene, is a major volatile component found in the essential oils of many plants [32, 33].Linalool has a pleasant floral scent therefore, widely used in cosmetics, especially fragrances and perfumes, in addition to pharmaceutical and food industries [34]. Many researchers have reported the antibacterial activity of linalool-rich essential oils. Previous studies indicated that linalool has anxiolytic, anti-cholesterol and antibacterial activity [35, 36].

α-Gurjunene was also included in the YY-EO profile and was previously detected by [37] among the volatile oil of *Melicope denhamii* leaves that exhibited both potent antimicrobial potential against both *Bacillus subtilis* and *E.coli,* and anticancer activity against Dalton's lymphoma ascites cells. Linalyl acetate the ester of linalool is also another volatile compound well known for antibacterial activity against foodborne bacteria such as *E. coli* and *Enterobacter cloacae* [31].

Monoterpenes as linalool generally show antimicrobial activity against Gram-positive bacteria but their effect depends on the amount of the compound present; at low concentrations, they can interfere with enzymes involved in the production of energy, and at higher concentrations, they can denature proteins [38] which might be mediating the observed antimicrobial effects in our study.

Generally speaking, Gram-negative bacteria are less susceptible to EOs than Gram-positive bacteria. The outer membrane of Gram-negative bacteria possesses hydrophilic lipopolysaccharides (LPS) acting as a barrier to macromolecules and hydrophobic compounds, thus providing increased tolerance to hydrophobic antimicrobial compounds such as those found in Eos [39]. However, the structure of the Gram-positive bacteria cell wall allows hydrophobic molecules to easily penetrate the cells and act on both the cell wall and within the cytoplasm.

Although YY-EO was tested against two isolates of the same strain, as in the case of *B. cereus,* S. *aureus, E. coli, P. aeruoginosa,* and *K. pneumonia*, results showed variability in terms of response to both tested antibiotics or YY-EO. This might be due to the breadth of genetic variations among species. Therefore, it is difficult to predict the susceptibility of microorganisms to EOs. Two isolates of the same strain type were variable in terms of response to the YY-EO; however, each strain was isolated from a different location. The environment affects the bacterial isolate response to antimicrobial agents, which might be due to evolutionary events that lead to the emergence of new resistance factors in pathogens [40].

YY-EO can be used as a potential natural preservative in cosmetics at a low concentration due to its antimicrobial properties, especially its effectiveness in controlling fungi, which is an important advantage of that preservative. It has been noticed that YY-EO applied exhibited high potential that YY-EOs exhibit high potential against Candida species, *C. tropicalis* MMCC12 and *C. parapsilosis* MMCC24 specifically.

Antimicrobial efficiency should be characterised by a broad spectrum of antimicrobial activity at a minimum concentration. Minimum inhibitory concentration results revealed that YY-EO was capable of inhibiting the growth of *S. aureus* MMCC22*, E. coli* MMCC24, and *C. tropicalis* MMCC12 up to 500 μg/ml. Such minimal effective concentration should be supported with cytoxicity data to indicate safety against allergy or sensitivity commonly occuring with EOs.

Gamma radiation provides an effective, heat-free alternative method for reducing or eliminating microbial contamination of EOs resulting from production procedures or handling. However, the effect of gamma radiation is quite variable, depending on both the type of EO and the dose applied.

In this study, YY-EO was subjected to various decontamination doses of gamma irradiation (0.5, 1, 1.5, and 6 kGy) to assess the effect of irradiation decontamination on the oil constituents. The antimicrobial susceptibility test was then re-conducted to evaluate the effect of the applied gamma doses on the antimicrobial potential of YY-EO upon radiation. Results suggest that gamma radiation could be used at definite doses as a decontamination tool without significantly affecting chemical structure or antimicrobial efficiency. YY-EO was then screened using FTIR. to confirm the consistency of the aromatic profile of the oil after irradiation.

Peaks in the "finger print region" exhibited no change at all tested doses. However, results of FTIR spectra of both non-irradiated and irradiated EO at different doses applied (0.5, 1, 1.5, and 6 kGy) exhibited one broad peak at wave numbers 2932, 2926, 2962, 2961, and 2927 cm1, respectively, assigned functional group C-H stretching-alkaline. A slight variation occurred, indicating a non-significant effect of gamma irradiation on EO chemical structure.

Up to the dose of 10 kGy, the gamma radiation barely changed the content of the essential oils until reaching the dose of 10.0 kGy. However, fewer monoterpenes were present in irradiated samples than in non-irradiated samples, but more sesquiterpenes and fatty acids were detected [41]. Preira and his colleagues examined the effects of gamma irradiation on the cytotoxicity and phenolic components of the traditional medicinal plants *Thymus vulgaris* L. and *Mentha piperita* L. In T. vulgaris and *Mentha piperita*, respectively, thirteen and fourteen phenolic compounds were found, and none of them were impacted by the irradiation dose utilized (10 kGy) [42]. The irradiation of 10 kGy had no effect on antioxidant properties of the essential oils [43]. The effects of gamma rays on oil properties in black cumin samples, reporting dose–effect responses at the level of quantitative microbial loading after irradiation with 2.5, 6, 8, and 10 kGy [44].

Recent studies on morphological changes provided evidence that different mechanisms are involved in the antibacterial activity of EOs. Disruption and permeabilization of bacterial membranes represent the best documented modes of action; the others are not fully understood and require more detailed studies. The results demonstrated by [45] showed that linalool treatment effectively inhibited the growth of *S. putrefaciens*, with complete destruction of the cell wall and membrane structure and function, promoting the leakage of intracellular substances and dysfunction of energy and metabolism.

Although the antifungal properties of EOs are well documented, the exact molecular mechanism is still unknown. The antifungal mode of action involving cytoplasmic membrane lesions caused by the disruption of sterol biosynthesis was documented in Candida, *Aspergillus flavus,* and *Botrytis cinerea* cells. Ergosterol is a specific sterol component of yeast cell membranes and is responsible for cell integrity and maintenance of cell vital functions, which is a main target of antifungal compounds [46]. On the other hand, the lipophilic properties of many components play a role in degrading the microbial plasma membrane, leading to the lysis of the hyphal wall [47]. It is assumed that other mechanisms, not involving membrane disruption, may also play a role in antifungal activity [47]. It is assumed that other mechanisms, not involving membrane disruption, may also play a role in antifungal activity.

Safety of the EO is a highly negotiable topic, especially being concentration-dependent. The safe concentration for using EOs is variable according to the type of oil, method, and frequency of application. The sensitivity of skin and allergic reactions due to the wrong application of oils are of high risk, especially with their potent cytoxic properties, making them a potential anticancer candidate.

Although YY-EO is one of the most highly exploited raw materials in the fragrance industry, it is also used in aromatherapy, household products, massage oils, and moisturising creams. However, cytotoxicity results revealed an unexpected potential cytotoxic effect against normal skin cells at 0.1%, which is a rather diluted dose.

One reported study highlighted the antitumor activity of a bioactive ingredient, β-caryophyllene (a terpene found in many plants, including ylang ylang), against prostate and breast cancer [30]. Also, β-caryophyllene induced apoptosis in lymphoma cell lines [47]. β-caryophyllene was detected in our aromatic profile results.

## Conclusion

Essential oils will always be thought of as a source of potential antimicrobials that researchers keep reaching out to. Few studies have approached the antimicrobial application of YY-EO on microbial cells. The present study detected the antimicrobial effect of YY-EO on different microbial strains, including Gram positive, Gram negative and yeasts, in terms of inhibition zones, in addition to the detection of morphological alterations that took place on three representative isolates following YY-EO application. In addition to the possible use of gamma irradiation as a decontamination tool that doesn’t affect Eos chemical composition if used at the right dose. 

## Limitations

Since YY-EO is commonly used as massage oil on human skin where natural flora lives, care should be taken concerning the routine application and applied concentration of the commercial YY-EO when used due to the potent antimicrobial and possible cytotoxic activity that might affect skin microbiota as well as normal skin cells. Many recent studies of eukaryotic cells have demonstrated that some essential oils may exert pro-oxidant and cytotoxic effects. Therefore, for the effective clinical use of essential oils, an evaluation of their cytotoxicity and the identification of the mechanisms affecting cell viability are required.

### Supplementary Information

Below is the link to the electronic supplementary material.

Supplementary file1 (DOCX 60 KB)

## Data Availability

The obtained data will be available upon request.
