# Supplementary material for: In-vitro Antimicrobial Study of Non/irradiated Ylang-ylang Essential Oil Against Multi Drug Resistant Pathogens with Reference to Microscopic Morphological Alterations
Source: Indian J Microbiol. 2023 Nov 10;63(4):621–31. doi: 10.1007/s12088-023-01122-4 (PMC10682331; doi:10.1007/s12088-023-01122-4)

**Table 1 : Volatiles’ profile composition of YY- EO**

| PK | Name | RT | Rel % 100 |
| --- | --- | --- | --- |
| 1 | ꞵ-Myrcene | 5.971 | 0.02 |
| 2 | Cyclohexene, 1-methyl-4-(1-methylethenyl)-, (S)- | 6.3887 | 0.03 |
| 3 | 1-Cyclohexene-1-methanol | 6.7377 | 0.03 |
| 4 | 2,4,6-Octatrienal | 7.3614 | 0.01 |
| 5 | Linalool | 7.6589 | 27.73 |
| 6 | Benzyl acetete | 8.4314 | 22.76 |
| 7 | Linalyl acetate | 9.0665 | 7.10 |
| 8 | α--ylangene | 9.8619 | 0.02 |
| 9 | trans-.beta.-Ocimene | 10.0793 | 0.02 |
| 10 | Copaene | 10.1366 | 0.27 |
| 11 | 2,6-Octadien-1-ol, 3,7-dimethyl-, acetate | 10.2624 | 0.50 |
| 12 | α-Gurjunene | 10.5543 | 17.69 |
| 13 | Caryophyllene | 10.6115 | 5.56 |
| 14 | cis-Thujopsene | 10.766 | 5.96 |
| 15 | Alloaromadendrene | 10.9834 | 3.82 |
| 16 | α-Guaiene | 11.0463 | 1.74 |
| 17 | (E,Z)-.alpha.-Farnesene | 11.2066 | 0.31 |
| 18 | R)-Cuparene | 11.3553 | 0.16 |
| 19 | δ-Cadinene | 11.4126 | 0.26 |
| 20 | 1,3-Cyclohexadiene, 1,5,5,6-tetramethyl- | 13.2093 | 0.11 |
| 21 | Valerena-4,7(11)-diene | 13.2493 | 0.05 |
| 22 | 2,4,6-Octatriene, 2,6-dimethyl- | 13.3237 | 0.10 |
| 23 | 1,3-Cyclohexadiene, 1,5,5,6-tetramethyl- | 13.4725 | 0.29 |
| 24 | Benzyl Benzoate | 13.5812 | 1.96 |
| 25 | Farnesol, acetate | 13.7242 | 0.64 |
| 26 | Benzylidene camphor | 14.6283 | 2.79 |
| 27 | Caryophyllene oxide | 15.0288 | 0.02 |
| 28 | Alloaromadendrene oxide-(1) | 15.3664 | 0.01 |
| 29 | Caryophyllene oxide | 15.7613 | 0.01 |
| 30 | Valerena-4,7(11)-diene | 15.9959 | 0.01 |
| 31 | Murolan-3,9(11)-diene-10-peroxy | 16.2018 | 0.01 |


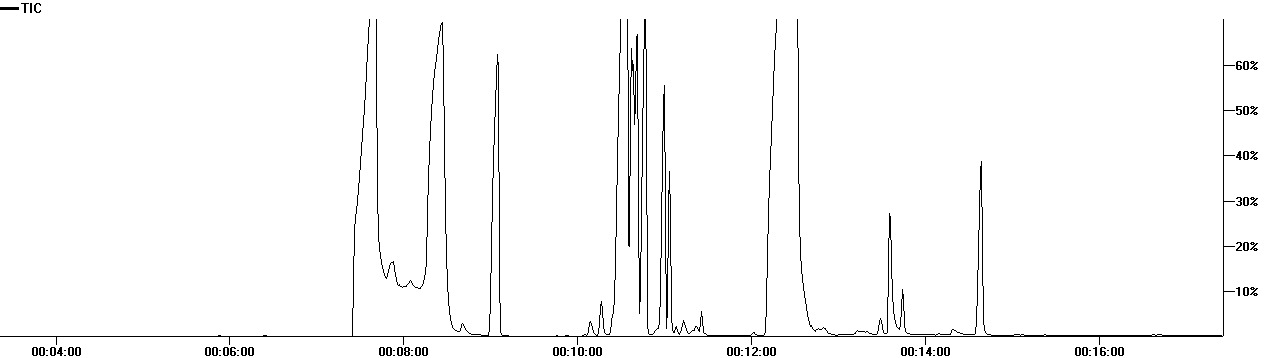

Supplement: Supplementary file 1 — Supplementary file1 (DOCX 60 KB) [file 12088_2023_1122_MOESM1_ESM.docx]
